# Factors associated with cognitive flexibility in people with opioid-use disorder: a pilot study

**DOI:** 10.3389/fpsyt.2024.1505391

**Published:** 2024-12-19

**Authors:** Paul S. Regier, Thais Costa Macedo de Arruda, Laura Sinko, Anne M. Teitelman, Anna Rose Childress

**Affiliations:** ^1^ Department of Psychiatry, Perelman School of Medicine, University of Pennsylvania, Philadelphia, PA, United States; ^2^ Psychology and Neuroscience Department, College of Liberal Arts, Temple University, Philadelphia, PA, United States; ^3^ Nursing Department, College of Public Health, Temple University, Philadelphia, PA, United States; ^4^ College of Nursing, Thomas Jefferson University, Philadelphia, PA, United States

**Keywords:** cognitive flexibility, neurocognition, opioid use disorder, impulsivity, social function

## Abstract

The ability to adapt to changing circumstances has strong survival value. Individuals with substance use disorders tend to get “stuck” over-responding to drug-reward signals and pursuing drugs despite negative consequences. A lack of flexibility may be tied to impairments in neurocognition, including learning, memory, and executive function. However, results are often mixed, potentially due to heterogeneity in factors such as mental health, personality traits, or prior adversity. This study aimed to identify which factors influence neurocognitive variations within the opioid use disorder (OUD) population. Based on prior literature, we hypothesized that individuals with OUD would show deficits (vs. controls) in one or more neurocognitive domains, and that these cognitive difficulties might be greater in individuals with other known contributors to impaired cognition. This pilot project included 32 individuals receiving medication for OUD and 15 non-substance using controls (NSC). Questionnaires assessed addiction and relapse risk factors, such as impulsiveness, social function, depressive symptoms, and childhood adversity. Neurocognitive performance was measured via the Penn Computerized Neurocognitive Battery (P-CNB), including tasks that probe attention, working memory, episodic memory, cognitive flexibility, and complex cognition, and was compared between the OUD and NSC groups. OUD participants (vs. NSCs) exhibited significantly lower performance on the conditional exclusion task (CET) (Accuracy: 1.11 vs. 2.38, p < 0.001) and the n-Back task (NBT) (F1 Scores: 83% vs. 95%, p < 0.001). Impulsiveness, social function, and depressive symptoms were highly inter-related; however, only higher impulsiveness (r = -.48, p = 0.006) and more social impairment (r = -.47, p = 0.007) significantly correlated with decreased CET (but not n-Back) performance. This pilot study suggests that working memory and cognitive flexibility are impaired in people with OUD and that impulsiveness and social function are key factors in cognitive flexibility impairments in people with OUD. These results may offer insights for larger-scale investigations and potential interventions to reduce relapse risk.

## Introduction

Drug overdose continues to be a major public health crisis, with approximately 75% of the 107,000 overdoses in 2022 caused by opioids (1). Non-fatal overdoses, estimated at ~600,000 based on emergency department visits (2), increase the risk of subsequent overdoses, including fatal ones (3–5). This data highlights the issue of individuals with substance use disorders tending to get “stuck,” over-responding to drug-reward signals and pursuing drugs despite negative consequences. Flexible decision-making requires the executive function system (6), and data on the impact of opioid-use disorder (OUD) on neurocognition indicate impairments in executive function domains (7, 8). Difficulties with executive function may contribute to an inability to make meaningful changes in drug-use behaviors (9). While studies show that executive function is generally impaired in people with OUD, factors other than opioid use, such as depression, impulsivity, social function, and prior adversity may also have an impact.

Studies investigating neurocognition in people with OUD indicate impairments in several cognitive domains (7, 8, 10, 11). For example, two meta-analyses found robust impairment in the cognitive domains of cognitive flexibility, memory, working memory, inhibition, and attention (7, 8). Individual studies indicate general cognitive deficits, though the specific domains affected vary. Two studies, using normative values as comparators, found that individuals with OUD exhibited the most impairment in learning and memory, but also in the domains of working memory, attention, and cognitive flexibility (11). In another study, people with OUD in different stages of treatment (e.g., detoxification, medications) generally scored lower on tasks measuring cognitive flexibility, working memory, attention, learning, and memory compared to non-substance-using controls (10). These findings underscore the importance of heterogeneity within the OUD population, which may be driven by different factors.

Data on factors that may contribute to more severe neurocognitive impairment are still emerging. Although mixed, studies suggest the importance of accounting for the potential impact of co-occurring issues on neurocognition, such as mental health (e.g., depression), behavioral characteristics (e.g., impulsiveness), general well-being (e.g., social life), polydrug use, and prior adversity (e.g., trauma). For example, one study found that depression contributed to lower cognitive performance, while polydrug use did not (10). In contrast, another found that polydrug use was associated with lower cognitive performance, while depression was not (12). Similarly, one study found about half of participants exhibited impairment in executive function, and that those with impairments had significantly less substance use but similar depression scores (13). Additionally, childhood adversity is often associated with increased risk for substance use disorders (14, 15), and emerging data suggest it may play a role in cognitive function (16), though this relationship remains understudied.

Impulsivity is a vulnerability factor for substance use disorder (SUD, 17, 18), and studies have shown that impulsiveness is higher in those with SUD including OUD (19, 20). Higher impulsivity scores correlate with more psychological problems (20), worse addiction treatment outcomes (21), and increased SUD severity (22). Emerging evidence suggests higher impulsivity may also be related to neurocognitive difficulties, such as lower cognitive flexibility (23). However, it is unclear whether impulsivity plays a role in neurocognitive difficulties in people with OUD.

Finally, social functioning is emerging as an important factor in SUD, particularly the ability to participate in social roles and activities. Previous studies have documented the general decline of social functioning in those with OUD (24, 25), such as more interpersonal conflict (25). Other studies, using validated measures, have found that social functioning is lower in individuals with OUD compared to controls (26, 27) and coincides with negative affective states (27). It is unclear whether social functioning is related to neurocognition in individuals with OUD.

While it has been established that neurocognition is generally impaired in people with OUD, it is unclear what factors might be related to this type of impairment. This knowledge gap limits our ability to develop targeted interventions to improve cognitive function in this population. This initial pilot study aimed to identify relevant factors, namely depression, impulsivity, social function, and prior adversity, that may be associated with neurocognition within the OUD population. Research in this area may inform the development of more effective, personalized treatment approaches that address both substance use and associated cognitive challenges.

## Methods

### Participants

Individuals with OUD (n=32) were recruited via flyers, referrals from a methadone clinic, and word of mouth. Inclusion criteria included: individuals who were on a stable dose of medication for OUD, aged 18-65, and able to read at an eighth-grade level. Exclusion criteria included: severe mental health issues (e.g., schizophrenia, current manic state, etc.), too impaired to complete the tasks, and pregnancy.

Neurocognitive data from non-substance-use controls (NSC, n=15) were obtained via a repository of de-identified data from pilot studies funded by the Penn Mental Health Aids Research Center. These individuals reported no alcohol or drug use on the Risk Assessment Battery (28). However, they did not complete several of the questionnaires administered to the OUD group (see below). The NSC group was matched to the OUD group on key demographic variables including age and sex to ensure comparability. All participants provided written informed consent, and the study protocol was approved by the University of Pennsylvania Institutional Review Board.

### Surveys/measures

Participants completed several questionnaires to capture data on demographics: age, sex, race, income, and education. Both groups also completed the Quick Inventory Depression Scale (QIDS) (29), see **Table 1**. Individuals with OUD additionally completed questionnaires on drug use, including opioids, stimulants, alcohol, and cannabis; social functioning via the PROMIS-Ability to Participate in Social Roles and Activities (PROM-SOC) (30); impulsiveness via the Barrat’s Impulsiveness Scale (BIS) (31); and prior adversity via the Extended Adverse Childhood Experiences (ACES) questionnaire (32). All questionnaires used were validated instruments with established psychometric properties.

**Table 1:** Demographics: average (± SEM).

	Overall (n=47)	NSC (n=15)	OUD (n=32)	Stats (NSC vs. OUD)
Age	46.0 (± 1.4)	46.7 (± 2.5)	45.7 (± 1.6)	t(45) = .36 (p = 0.42)
Sex (%female)	72.3%	60.0%	78.1%	χ^2^ = 1.68 (p = .30)
Race (%Black)	44.7%	33.3%	50.0%	χ^2^ = 1.15 (p = .36)
**QIDS**	**12.7 (± 1.3)**	**4.1 (± 0.8)**	**16.5 (± 1.3)**	**t(44) = 5.95 (p < 0.001)**
**Education (Some college or more) (NSC = 13)**	**34.1%**	**7.7%**	**45.2%**	**χ^2^ = 5.72 (p = 0.02)**
**Income (25k or more) (NSC = 8)**	**25%**	**75%**	**12.5%**	**χ^2^ = 13.33 (p = 0.001)**

**Bold** lettering highlights significant differences.

### Penn computerized neurocognitive battery

Both groups completed the Penn Computerized Neurocognitive Battery (CNB) (33). This battery included tasks to measure episodic memory via the Face (FMT) and Word (WMT) Memory Tasks; working memory via the letter n-Back task (NBT); cognitive flexibility via the Conditional Exclusion Task (CET); complex cognition via the Spatial Line Orientation Task (LOT); and emotion recognition via the Emotion Recognition Task (ERT). The CNB also included tasks to evaluate proficiency in using a computer mouse as well as a motor task (i.e., quickly pressing the space bar) as control factors. Details and factor testing of the tasks have been reported elsewhere (34). All tasks were administered in a standardized order, and participants were given breaks as needed to minimize fatigue.

The primary performance measure for FMT, WMT, ERT, and LOT was the number of correct responses. The primary measure for NBT was the F1 score, calculated using the formula, (2*TP)/(2*TP+FP+FN), where TP = true positives, FP = false positives, and FN = false negatives. For the CET, the primary measure was an accuracy score, calculated by (Number of categories achieved + 1) * CR/(CR+ER), where CR = correct responses and ER = errors.

### Analysis

Potential differences between groups were assessed via independent t-tests (for continuous variables) and χ^2^ (for nominal variables), with a threshold of *p* < 0.05, FDR corrected. Effect sizes (Hedge’s *g*) are reported for significant differences. Pearson correlations were used to examine relationships between questionnaire scores and cognitive performance within the OUD group. All analyses were performed using SPSS (v28, 2021).

## Results

### Demographics/health

There were forty-seven total individuals included in the analysis, with 15 in the NSC group and 32 in the OUD group. Average age was 46 (OUD: 45.7 (±1.6); NSC: 46.7 (±2.5); *t*(45) = .36, *p* = 0.42). There were 72.3% of individuals who were female (OUD: 78.1%; NSC: 60%; χ^2^ = 1.68, *p* = 0.30) and 44.7% who were African American (OUD: 50%; NSC: 33.3%; χ^2^ = 1.15, *p* = 0.36), and no significant differences between the two groups. The OUD group had significantly higher QIDS scores compared to the NSC group (OUD: 16.5 (±1.3); NSC: 4.1 (±0.8); *t*(44) = 5.95, *p* < 0.001, *g* = 1.9).

For the OUD group, additional information was collected. Regarding type of medication participants were taking for OUD, twenty-two (73%) reported methadone, 7 (23%) reported Suboxone, and 1 (3%) reported Naltrexone. Approximately 44% had some college or more (vs. completing high school or less); 40% were experiencing current houselessness; and 84% had at least one adverse childhood experience. Regarding drug use, the OUD group had used opioids for an average of 14 years, with approximately 47% reporting fentanyl use. Approximately 44% reported alcohol use; 38% reported cannabis use; 47% reported stimulant use; and 56% reported use of more than 1 drug.

A subgroup of NSCs completed questionnaires about education (n=13) and income (n=8), both of which differed from the OUD group. There were only 1/13 of NSCs who had some college or more (vs. completing high school or less) compared to 14/32 of the OUD group who had some college or more (χ^2^ = 5.72, *p* = 0.02). In contrast, 6/8 of NSCs made more than 25k (vs. less than 25k) compared to only 4/32 of OUD who made more than 25k (χ^2^ = 13.33, *p* = 0.001). Too few numbers in the NSC group prevented further investigation of the effect of income on CNB performance.

### CNB performance

**Figure 1:**
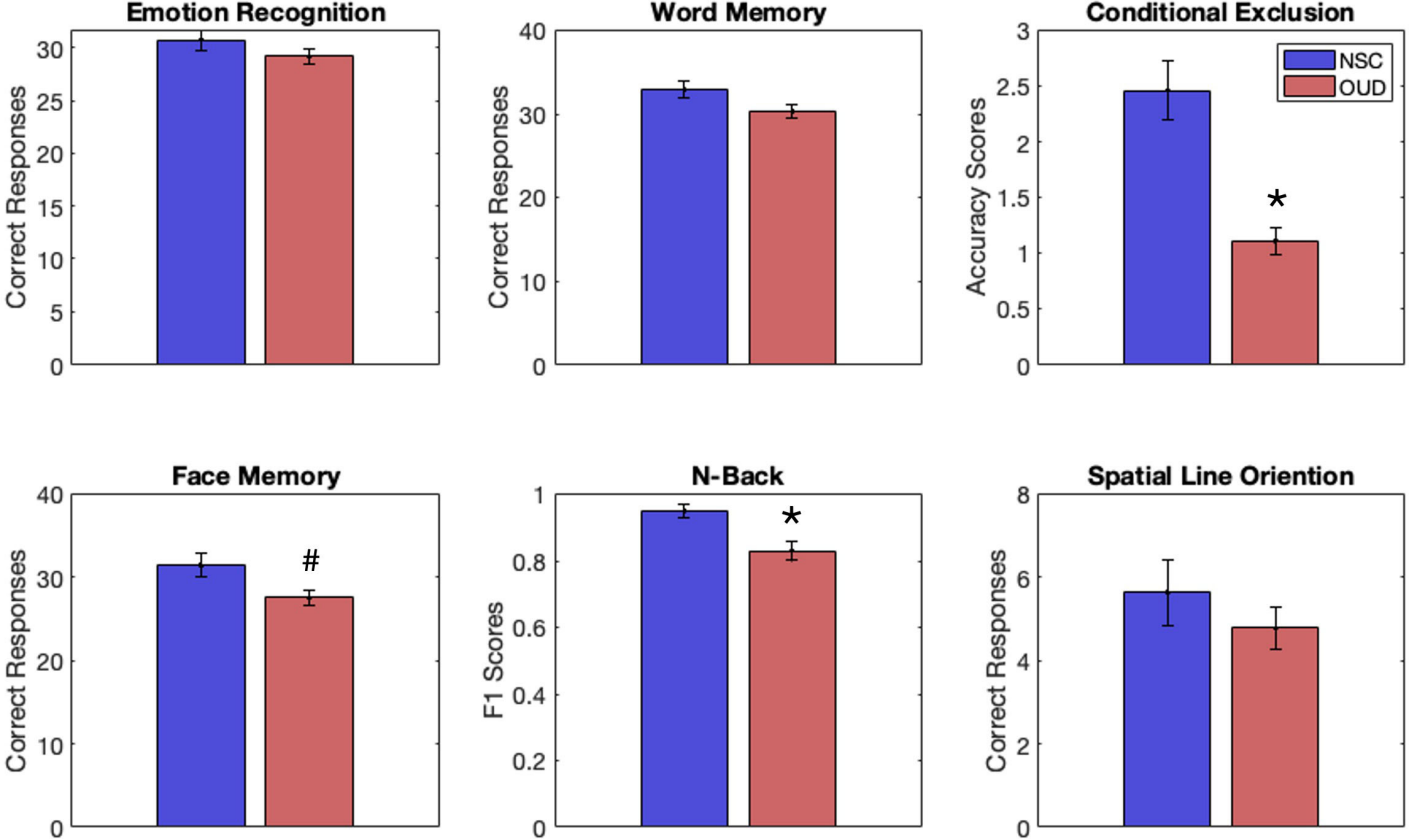
Behavioral performance scores for individuals with opioid-use disorder (OUD, red) and non-substance-using controls (NSCs, blue) on tasks from the Penn Computerized Neurocognitive Battery, including emotion recognition, word memory, conditional exclusion (CET), face memory (FMT), n-back (NBT), and spatial line orientation. Individuals with OUD had significantly lower scores (vs. NSCs) on the CET and NBT, FDR correction. An uncorrected result suggested lower scores for OUD (vs. NSCs) on the FMT. *p < 0.05 (FDR corrected). ^#^p < 0.05 (uncorrected).

Results, FDR corrected, show that the OUD group had significantly less accuracy on the CET (*t*(45) = 5.26, *p* < 0.001) and lower F1 scores on the NBT (*t*(43) = 3.13, *p* = 0.003, *g* = 1.6). Uncorrected results showed fewer correct responses on the FMT (*t*(45) = 2.32, *p* = 0.03, *g* = .72). There was a trend towards fewer correct responses on the WMT (*t*(45) = 1.86, *p* = 0.07). See **Figure 1** and **Table 2** for full statistics and results. Correcting for differences in QIDS did not change results for the CET (*p* = 0.001) or FMT (*p* = 0.043) but made NBT results insignificant (*p* = 0.19).

**Table 2:** CNB results – correct responses/accuracy (± SEM).

	Overall	NSC (n=15)	OUD (n=32)	Stats
Emotion Recognition	29.7 (± 0.59)	30.7 (±.93)	29.2 (±.75)	t(45) = 1.20 (p = 0.24)
Word Memory	31.1 (± 0.67)	32.9 (± 1.05)	30.3 (±.83)	t(45) = 1.86 (p = 0.07)
**Conditional Exclusion^1^**	**1.54 (± 0.15)**	**2.46 (±.27)**	**1.11 (±.12)**	**t(45) = 5.26 (p < 0.001)**
**Face Memory**	**28.8 (± 0.81)**	**31.47 (± 1.45)**	**27.59 (±.92)**	**t(45) = 2.32 (p = 0.03)**
**N-Back^2^**	**0.87 (± 0.02)**	**.95 (±.02)**	**.83 (±.03)**	**t(43) = 3.13 (p = 0.003)**
Spatial Line Orientation	5.04 (± 0.43)	5.64 (±.80)	4.78 (±.50)	t(44) = .93 (p = .36)

^1^Accuracy Score; ^2^F1 Scores; **Bold** lettering highlights significant differences.

The groups did not differ on reaction times. Uncorrected results showed a trend towards differing reaction time on the WMT (*t*(45) = 1.8, *p* = 0.08). See **Table 3** for full statistics and results on reaction times.

**Table 3:** CNB results – reaction times (± SEM).

	Overall	NSC (n=15)	OUD (n=32)	Stats
Emotion Recognition	2523 (± 119)	2498 (± 321)	2534 (± 95)	t(45) = .14 (p = 0.89)
Word Memory	1881 (± 70)	1701 (± 101)	1965 (± 89)	t(45) = 1.8 (p = 0.08)
Conditional Exclusion	3047 (± 215)	3010 (± 563)	3064 (± 183)	t(45) = .12 (p = 0.91)
Face Memory	1845 (± 60)	1857 (± 124)	1840 (± 68)	t(45) = .13 (p = .89)
N-Back	601 (± 25)	548 (± 46)	627 (± 28)	t(44) = 1.5 (p = .13)
Spatial Line Orientation	16430 (± 1661)	15673 (± 3254)	16761 (± 1950)	t(44) = .3 (p = 0.77)

Times are listed in milliseconds.

### Correlations of CNB performance with mental health and behavioral variables

There were several significant correlations between measures on mental health symptoms and behavioral variables. The QIDS and BIS were positively correlated (r = .51, *p* = 0.003); the QIDS and PROM-SOC were inversely correlated (r = -.51, *p* = 0.003); and the BIS and PROM-SOC were inversely correlated (r = -.74, *p* < 0.001).

**Figure 2:**
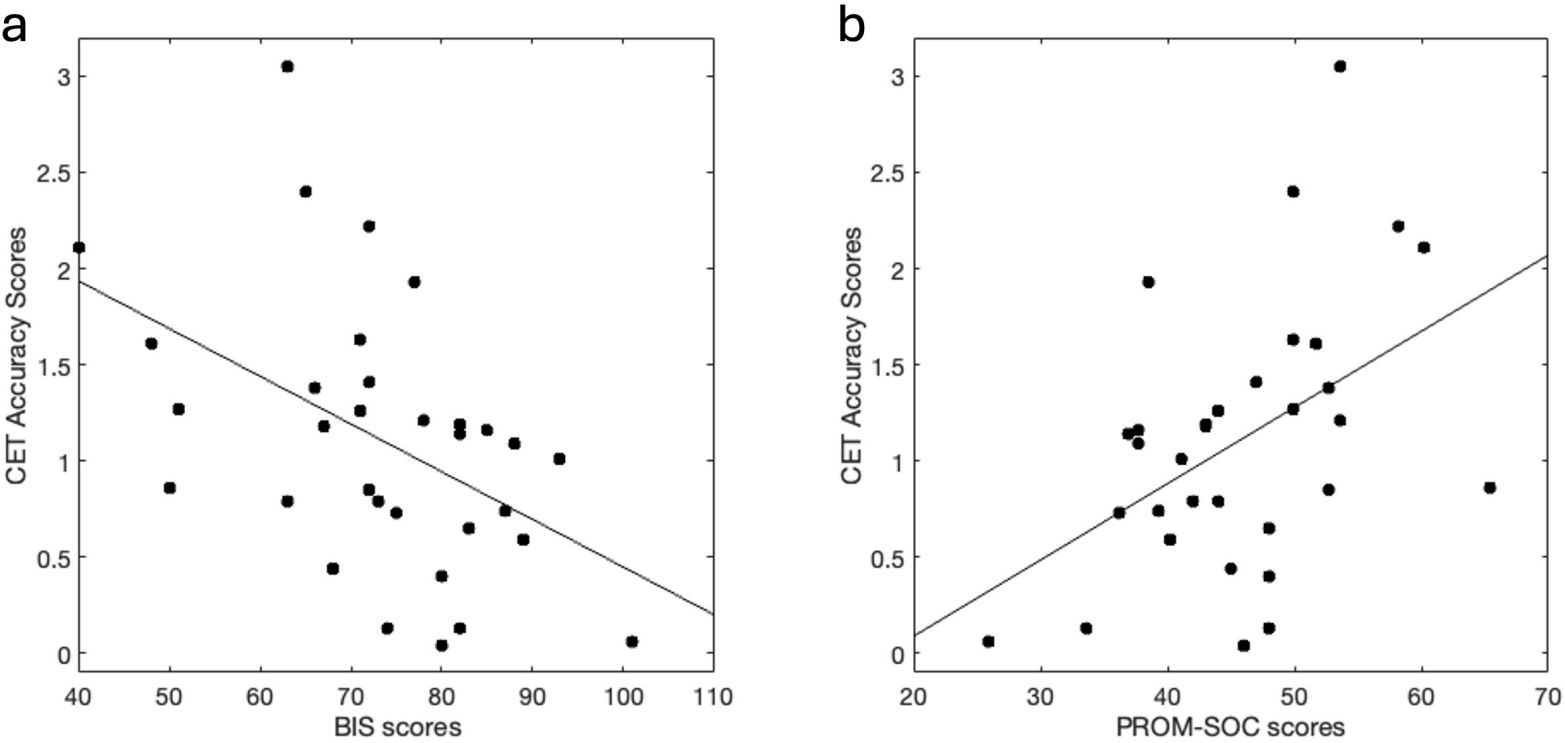
Cognitive flexibility performance correlates with Impulsiveness and Social Function. **(A)** Higher impulsiveness scores (via BIS) correlated with lower cognitive flexibility (via CET) performance (r = -.47, p = 0.006). **(B)** Higher social functioning scores (via PROM-SOC) correlated with better cognitive flexibility performance (r = .46, p = 0.007). BIS, Barrat’s impulsiveness scale; CET, Penn Conditional Exclusion Task; PROM-SOC, PROMIS- Ability to Participate in Social Roles and Activities.

Performance on the CET was inversely correlated with BIS scores (r = -.47, *p* = 0.006), in that lower performance was associated with more impulsiveness (**Figure 2A**). Correlations were conducted to explore the relationship of CET performance with BIS subscales (**Supplementary Table S2**). Performance on CET was positively correlated with PROM-SOC scores (r = .46, *p* = 0.007) (**Figure 2B**), in that better performance was associated with higher social functioning. There were no significant correlations between mental health or behavioral variables with NBT or FMT scores.

Prior adversity and QIDs did not associate with CET (**Supplementary Table S1**). Other factors such as polydrug use, education, age, and sex also did not associate with CET performance (**Supplementary Table S1**).

## Discussion

As expected, individuals with OUD (vs. NSCs) demonstrated lower performance on several neurocognitive tasks, specifically those measuring cognitive flexibility and memory. The OUD group showed lower accuracy scores on the CET and lower F1 scores on the NBT, and data suggests number of correct responses on the FMT were lower, as well. Better performance on the CET, but not NBT or FMT, was associated with lower impulsiveness and higher social functioning. Notably, no mental health and/or behavioral variables were associated with NBT or FMT (see **Supplementary Table S1**). Results on the NBT were not significant after controlling for depression symptoms.

The significantly higher QIDS scores in the OUD group highlight the comorbidity of depression and OUD. This finding is particularly relevant as depression symptoms may exacerbate cognitive deficits and complicate treatment outcomes. The fact that NBT results became insignificant after controlling for depression symptoms suggests a complex interplay between working memory, depression, and OUD. Future research should aim to disentangle these relationships to better inform treatment strategies that address both cognitive deficits and depressive symptoms in OUD patients.

The primary significance from this study was the stark difference between OUD and NSC groups on cognitive flexibility measured via the CET. In addition to significantly lower accuracy scores, the OUD group made almost three times the number of perseverative errors compared to NSCs (OUD: 31.4 [± 2.23]; NSC: 11.2 [± 1.89], *p* < 0.001). Furthermore, while participants can complete up to three categories of rules based on the parameters, and the OUD group, on average, only completed 1.5 categories; and the NSC group completed, on average, 2.5 categories, a significant difference (*p* = 0.001).

The associations between CET performance and the variables of impulsiveness and social function suggest both vulnerability and protection concerning difficulties with cognitive flexibility, respectively. Being able to receive, process, adapt and adequately respond to new information is critical in social interactions (35), with the positive correlation between the two indicating that strong cognitive flexibility skills can support social functioning. Conversely, the inability to control impulses can hinder an individual’s capacity to process and respond to new information adequately (36). Interestingly, these variables were interrelated, suggesting that there may be an even more complex interplay between cognitive flexibility, impulsivity, and social functioning.

Based on previous research, it seems possible that impulsiveness is a predisposing factor driving primary differences, as pre-treatment levels determine clinical outcomes (21). One possibility is that pre-existing neurocognitive difficulties are partially driven by impulsiveness, and subsequently exacerbated by opioid exposure (37, 38). Some of the defining features of addiction include difficulties related to social situations at home, work, and school (39), and recent studies underscore the importance of flexibility in social situations (40). Therefore, cognitive flexibility difficulties exhibited by the OUD group may be further related to social functioning challenges.

The complex relationships between cognitive flexibility, impulsivity, and social functioning may help illuminate novel paths to understanding the challenges faced by those with OUD. While previous studies have identified associations between social function, neurocognition, and outcomes in those with severe mental illness (41), less is known about these connections in individuals with addiction. The current study reveals how cognitive flexibility may be a key aspect in understanding behavioral challenges in OUD. The strong correlation between social functioning and cognitive flexibility described here suggests a significant relationship, warranting further investigation into whether this association influences clinical outcomes and how impulsivity may be a predisposing or risk factor.

### Treatment implications

Emerging research suggests cognitive difficulties might be improved by various means (42). For example, one study found that cognitive deficits improved with repeated testing and with increased abstinence from opioids (43). Some data suggests cognitive training improves performance on neurocognitive tasks, though it is unclear how well these improvements translate into real-world situations (44).

Executive function is predominantly regulated by cortical, particularly prefrontal cortical (PFC), regions (45), leading to downstream inhibition of subcortical regions (46); thus, bolstering PFC regions directly might improve neurocognition generally (47). Indeed, some methods, such as transcranial magnetic stimulation of lateral PFC regions have been successful in improving executive function (48, 49). Some studies have reported on the importance of the left (vs. right) dorsolateral PFC in reducing drug-seeking behaviors (50), suggesting the inhibitory impact of that area.

Pharmacological effects may also be helpful in improving both neurocognition and impulsivity, especially considering that cognition may not improve while receiving a medication for OUD (51). Psychedelics as therapeutic agents have had a resurgence in the past two decades, and studies show promise of psychedelic-assisted treatment in treating mental health issues, such as depression (52), trauma (53), and substance-use disorder (54, 55). In addition, psychedelics studies show that neurocognition, particularly cognitive flexibility, is improved post psychedelic-assisted treatment (56), which is thought to be due to changes occurring at the neural level (e.g., neuroplasticity) (57, 58).

### Clinical outcomes

Studies show the importance of cognition in clinical outcomes. For example, impairments in cognition have been tied to treatment non-adherence, often in older adults (59–61); impairment that are typically accelerated in those with OUD (62). and difficulties with decision-making, cognitive flexibility, and inhibition have been associated with increased drug-seeking behaviors and relapse (63–65). Thus, treatments to improve neurocognition could translate to better clinical outcomes.

The clinical significance of these findings cannot be overstated. Healthcare providers working with individuals with OUD should be aware of the potential cognitive deficits, particularly in cognitive flexibility and working memory. Routine cognitive assessments could help tailor treatment plans to individual needs. Moreover, the strong associations between impulsivity, social function, and cognitive flexibility suggest that a multifaceted treatment approach addressing these interconnected factors may be most effective in improving outcomes for individuals with OUD.

### Limitations

This was a pilot study with a relatively small clinical sample size, with primarily a female population and high trauma exposure. In addition, the comparator group was obtained via a repository of data from different pilot studies. Therefore, data collection methods may have varied, and several questionnaires were not collected in the NSC group. A follow-up study is in progress that aims to triple the OUD sample size as well as collect the same data from matched NSCs. In addition, demographic differences between the OUD and NSC groups, particularly in income levels, may have influenced cognitive performance. Future studies should aim to control for these variables to isolate the specific effects of OUD on cognitive function. Finally, the limited dataset also limited the statistical approaches that could be applied.

### Conclusions

This pilot study suggests that working memory and cognitive flexibility are impaired in people with OUD and that impulsiveness and social function are key factors in cognitive flexibility impairments in OUD. The results may offer insights for larger-scale investigations and potential interventions to reduce relapse risk. Future research should focus on developing and testing interventions targeting cognitive flexibility and impulsiveness in OUD, as well as exploring the potential role of social function in treatment outcomes. By addressing these cognitive and psychosocial factors in tandem with traditional OUD treatments, we may be able to significantly improve outcomes for individuals struggling with opioid addiction.

## Data Availability

The raw data supporting the conclusions of this article can be made available by the authors, without undue reservation.
