# Supplementary material for: Factors associated with cognitive flexibility in people with opioid-use disorder: a pilot study
Source: Front Psychiatry. 2024 Dec 19;15:1505391. doi: 10.3389/fpsyt.2024.1505391 (PMC11693614; doi:10.3389/fpsyt.2024.1505391)
Supplement: Supplementary file 1 [file DataSheet1.docx]

Supplemental Table 1. Regression results in the opioid group only, measuring the association of several factors with CET performance (OUD group only).

|  | R Squared | P Value |
| --- | --- | --- |
| Age | 0.01 | 0.62 |
| Sex | 0.02 | 0.42 |
| Race | 0.02 | 0.48 |
| Education | < 0.00 | 0.85 |
| Opioid Use (years) | 0.09 | 0.12 |
| Polydrug use | 0.08 | 0.65 |
| Prior Adversity | 0.06 | 0.74 |
| Depression (QIDS) | 0.04 | 0.28 |
| Social Function (PROM-SOC) | 0.21 | <0.01 |
| Impulsiveness (BIS) | 0.22 | <0.01 |

Supplemental Table 2. Subscales of the Barratt’s Impulsiveness Scale Associated with CET performance (OUD group only)

|  | R Squared | P Value |
| --- | --- | --- |
| BIS-ATTN | 0.26 | 0.002 |
| BIS-MOT | 0.09 | 0.091 |
| BIS-NP | 0.15 | 0.026 |

Supplemental Table 3. Correlation Matrix of Performance on CNB (OUD group only)

|  | Emotion Recognition | Word Memory | Conditional Exclusion | Face Memory | N-Back |
| --- | --- | --- | --- | --- | --- |
| Emotion Recognition | 1 | - | - | - | - |
| Word Memory | R = .298 (p = 0.10) | 1 | - | - | - |
| Conditional Exclusion | R = .367 (p = 0.04) | R = .071 (p = 0.70) | 1 | - | - |
| Face Memory | R = .688 (p < 0.001) | R = .591 (p < 0.001) | R = .249 (p = 0.17) | 1 | - |
| N-Back | R = .248 (p = 0.19) | R = .520 (p = 0.003) | R = .043 (p = 0.82) | R = .449 (p = 0.01) | 1 |
| Spatial Line Orientation | R = .308 (p = 0.31) | R = .146 (p = 0.43) | R = .340 (p = 0.06) | R = .368 (p = 0.04) | R = .465 (p = 0.01) |
